# Clinical characteristics and preventable acute care spending among a high cost inpatient population

**DOI:** 10.1186/s12913-016-1418-2

**Published:** 2016-05-04

**Authors:** Paul E. Ronksley, Daniel M. Kobewka, Jennifer A. McKay, Deanna M. Rothwell, Sunita Mulpuru, Alan J. Forster

**Affiliations:** Department of Community Health Sciences, University of Calgary, 3330 Hospital Drive NW, Calgary, T2N 4N1 AB Canada; Department of Medicine, University of Ottawa, Ottawa, ON Canada; Department of Epidemiology and Community Medicine, University of Ottawa, Ottawa, ON Canada; Performance Measurement, The Ottawa Hospital, Ottawa, ON Canada; Department of Clinical Epidemiology, Ottawa Hospital Research Institute, Ottawa, ON Canada

**Keywords:** Administrative data, Ambulatory care sensitive conditions, Healthcare cost, Hospitalization

## Abstract

**Background:**

A small proportion of patients account for the majority of health care spending. The objectives of this study were to explore the clinical characteristics, patterns of health care use, and the proportion of acute care spending deemed potentially preventable among high cost inpatients within a Canadian acute-care hospital.

**Methods:**

We identified all individuals within the Ottawa Hospital with one or more inpatient hospitalization between April 1, 2010 and March 31, 2011. Clinical characteristics and frequency of hospital encounters were captured in the information systems of the Ottawa Hospital Data Warehouse. Direct inpatient costs for each encounter were summed using case costing information and those in the upper first and fifth percentiles of the cumulative direct cost distribution were defined as extremely high cost and high cost respectively. We quantified preventable acute care spending as hospitalizations for ambulatory care sensitive conditions (ACSC) and spending attributable to difficulty discharging patients as measured by alternate level of care (ALC) status.

**Results:**

During the study period, 36,892 patients had 44,066 hospitalizations. High cost patients (*n* = 1,844) accounted for 38 % of total inpatient spending ($122 million) and were older, more likely to be male, and had higher levels of co-morbidity compared to non-high cost patients. In over half of the high cost cohort (54 %), costs were accumulated from a single hospitalization. The majority of costs were related to nursing care and intensive care unit spending. High cost patients were more likely to have an encounter deemed to be ambulatory care sensitive compared to non-high cost inpatients (6.0 versus 2.8 %, *p* < 0.001). A greater proportion of inpatient spending was attributable to ALC days for high cost versus non-high cost patients (9.1 versus 4.9 %, *p* < 0.001).

**Conclusions:**

Within a population of high cost inpatients, the majority of costs are attributed to a single, non-preventable, acute care episode. However, there are likely opportunities to improve hospital efficiency by focusing on different approaches to community based care directed towards specific populations.

**Electronic supplementary material:**

The online version of this article (doi:10.1186/s12913-016-1418-2) contains supplementary material, which is available to authorized users.

## Background

In Canada, total health care expenditures have doubled in the past decade and are forecasted to exceed $200 billion by 2014 [1]. While improving patient care and reducing costs remain a priority within the Canadian health care system, the fact that actual spending is distributed unevenly across individuals in the population creates an opportunity to direct improvement efforts to a limited group of patients with a potential high benefit. It has been estimated that the top 5–10 % of health care users account for over 50 % of health care utilization and cost [2–6]. Furthermore, approximately 40 % of all health spending is for hospital care which is similarly skewed towards the so-called ‘high-user’ [1, 5, 6]. For these reasons, it is logical to assess hospital spending in this patient population.

While a number of prior studies have characterized high cost hospital patients [3–12], there are limited data to direct efforts in a single-payer publicly funded health system. Many of these studies have focused on patients with specific chronic medical conditions, elderly populations, or certain insurance beneficiaries within the United States limiting the usefulness and generalizability of their findings to an entire health system. Furthermore, few studies have assessed the hospital resources most commonly used during high cost encounters; rather they look at overall costs. This limits our understanding of how costs are accumulated, the specific resources that contribute to overall costing estimates, and the proportion of inpatient spending deemed potentially preventable among high cost inpatients. While the latter has recently been studied by Joynt et al., within an American Medicare population, it is unclear how these results translate to other settings with different funding models, like we have in Canada [5].

It is critical to generate an evidence-base on this topic. Many hospitals and health systems are faced with financial pressures. To improve care to these high cost patients, many have called for re-distribution of resources to provide better disease management [13, 14]. These efforts are predicated on the assumption that at least some of the spending on these users is due to poor coordination of care. Without understanding the subset of patients, their health concerns and the treatments that are costing the system disproportionate amounts, then it is unclear whether these efforts will bear fruit. For this reason, we used detailed clinical and administrative data to characterize high cost patients within a Canadian tertiary-care teaching facility and determine the proportion of acute care spending deemed potentially preventable.

## Methods

### Data sources and study population

This was a retrospective observational study using The Ottawa Hospital Data Warehouse. The Ottawa Hospital is a tertiary-care teaching facility with two acute-care campuses containing 1100 beds. It is the largest referral centre for a population of 1.1 million people. The Data Warehouse is a relational database containing information from several of The Ottawa Hospital’s information systems including the patient registration system, clinical data repository, case costing system, and patient abstracts for multiple encounter types. Within these data sources, we identified all individuals (regardless of age) with one or more inpatient hospitalizations between April 1, 2010 and March 31, 2011. We excluded hospital events with admission dates that fell outside of the defined timeframe. However, events with discharge dates that fell outside of this timeframe were included.

### Identification of high cost patients

Direct costs for each inpatient encounter were identified within the case costing system of the Ottawa Hospital Data Warehouse. The case costing system links financial, clinical, and patient activity information stored within information systems of the data warehouse to define ‘intermediate products’ (e.g. nursing time, medications, laboratory tests, surgical material, etc.). The direct and indirect costs for each intermediate product used within an encounter are then summed for each patient. The Ottawa Hospital employs a standardized case costing methodology developed by the Ontario Case Costing Initiative [15], and is based on the Canadian Institute for Health Information (CIHI) Management Information Systems guidelines and the Ontario Healthcare Reporting Standards [16]. The primary purpose of case costing standards is to ensure comparability across Canadian hospitals. Costs were summed for patients with multiple hospitalizations during the defined time frame. Using the cumulative direct cost distribution, we then defined patients in the 1^st^ percentile of total cost as extremely high cost patients and those in the upper 5^th^ percentile as high cost patients.

### Clinical characteristics and resource utilization

We used information from the patient registry file and hospital abstracts to measure clinical and demographic characteristics. Using the first (index) hospitalization for each patient within the study timeframe, we measured age, sex, marital status, and comorbidity. Hospital discharge abstracts data were used to determine the number of hospital encounters during the study period and to identify specific encounter-level characteristics including admission and discharge date, admission type (elective, newborn, urgent, emergency, day surgery admits), most responsible diagnosis, procedure codes, length of stay (acute, alternate level of care (ALC) and intensive care unit (ICU) days), and comorbidity defined using the Elixhauser comorbidity index and derived Elixhauser comorbidity score [17, 18]. Coded inpatient information within the Ottawa Hospital Data Warehouse employs the same data quality standards as the CIHI Discharge Abstracts Database [19] and is based on the International Statistical Classification of Diseases and Related Health Problems, 10^th^ revision – Canada (ICD-10-CA).

In-hospital resource utilization was captured using individual direct cost details within the case costing system. These include 11 resource-specific categories based on Ontario Quality Based Procedures Groupings (e.g. Nursing costs, laboratory costs, pharmacy costs, operating room costs (Additional file 1)). The relative proportion of total cost attributable to each resource category were measured by summing category costs and dividing them by the total direct hospital costs for high cost and extremely high cost groups.

### Identification of preventable acute care spending

Preventable acute care spending was measured in two different ways. First, we used the Canadian Institute for Health Information ambulatory care sensitive condition (ACSC) indicator algorithm to identify potentially preventable hospitalizations. This algorithm defines potentially preventable hospitalizations as those related to health conditions for which good outpatient care can likely prevent the need for hospitalization [20]. The use of the ACSC indicator has been recognized internationally as a measure of adequacy of ambulatory and primary health care performance [21–23]. Within Canada, there is a focus on seven conditions: hypertension, diabetes, angina, asthma, chronic obstructive pulmonary disease (COPD), epilepsy, and heart failure/pulmonary edema derived from diagnosis and procedure codes identified within the hospital discharge abstracts data (Additional file 2). Second, we quantified spending attributable to difficulty discharging patients as measured by alternate level of care (ALC) status. ALC status refers to patients who no longer need acute care services but continue to use hospital resources while they wait to be discharged to a more appropriate setting [24]. Standardized collection of ALC data is available within hospital discharge abstracts and is used to isolate acute and non-acute components of a hospital encounter.

### Analysis

Patient characteristics were described using proportions, means (standard deviation (SD)), and medians (inter-quartile range (IQR)) where appropriate. Measures were compared between high cost and non-high cost groups using the two definitions described above. Next, we calculated the number of hospitalizations (categorized as 1, 2, 3, ≥4 encounters), median days in hospital (total and acute days), the proportion of patients with an ICU admission, and the median total ICU days among those with an ICU encounter. Similarly, the proportion of patients with at least one ALC day was calculated, and the median total ALC days among those with an ALC component to their encounter. In-hospital mortality and proportion with a 30-day all-cause readmission to hospital were also calculated across high cost categories. Admission type and the most frequent most responsible diagnoses were reported for high cost groups with a single high cost encounter. Concentration of direct inpatient spending was calculated across high cost groups and was reported as a percentage of total direct inpatient spending as well as the average direct cost per patient. The proportion of total costs incurred within each hospital resource category and costs attributable to ALC days was also measured across high cost groups.

We calculated the proportion of patients with a hospital encounter deemed to be potentially preventable using the ACSC indicator algorithm for high cost (upper 5^th^ percentile) and non-high cost groups. Based on the algorithm inclusion/exclusion criteria, estimates were calculated among patients <75 years of age, and excluded those that died prior to discharge, and newborn admissions. Overall and condition-specific estimates were reported across high cost status. Given limitations in sample size, this analysis was not conducted using the extremely high cost (upper 1^st^ percentile) cut-point.

Logistic regression was used to identify independent predictors of ALC status (defined as one or more ALC days within a hospital encounter) among high cost patients (upper 5^th^ percentile). Initially, univariate odds ratios (OR) were calculated for socio-demographic factors, comorbidity, and clinical factors related to the hospital encounter. A multivariate model was then developed based on significant predictors of ALC status and was reduced using backwards elimination techniques. As the analysis occurred at the encounter level, robust standard errors were calculated using the Huber-White “sandwich estimator” to account for clustering of hospitalizations at the patient level [25, 26]. Further analyses were not conducted using the extremely high cost (upper 1^st^ percentile) cut-point based on sample size limitations. For all statistical tests, *p* < 0.05 was considered statistically significant. All analyses were conducted using STATA 13.0 statistical software (Statacorp, College Station, TX). This study was approved by the Ottawa Health Sciences Network Research Ethics Board and granted waiver of patient consent.

## Results

### Clinical characteristics and resource utilization among high cost inpatients

Between April 1, 2010 and March 31, 2011 a total of 36,892 patients had 44,066 hospitalizations. Cumulative direct costs of ≥ $83,000 and ≥ $33,000 defined the upper 1 and 5 percentiles respectively. Based on these cost cut-points, 369 patients constituted the extremely high cost group and 1844 patients the high cost group. In general, both high cost groups were older, more likely to be male, and had higher levels of comorbidity compared to their non-high cost comparators (Table 1). In particular, hypertension (uncomplicated), diabetes (with or without complications), cardiac arrhythmias, cancer, congestive heart failure, and COPD were the most prevalent conditions amongst high cost patients.

**Table 1:** Patient characteristics

		Top 1 %		Top 5 %	
Variable	All	High Cost	Non High Cost	High Cost	Non High Cost
	(*n* = 36,892)	(*n* = 369)	(*n* = 36,523)	(*n* = 1,844)	(*n* = 35,048)
Age, yrs median (IQR)	46 (26–68)	61 (47–74)	45 (26–68)	66 (52–78)	44 (25–67)
Category, %					
- 0–18	18.9	7.9	19.0	6.0	19.5
- 19–45	31.2	15.7	31.3	12.0	32.2
- 46–69	27.0	45.5	26.8	39.6	26.3
- 70–79	11.4	17.3	11.3	21.3	10.9
- 80+	11.6	13.6	11.6	21.1	11.2
Male, %	39.5	57.5	39.3	54.4	38.7
Marital Status, %					
- Married/Common-law	51.3	49.3	51.3	53.1	51.2
- Single	31.5	29.8	31.5	22.2	31.9
- Separated/Divorced/Widowed	12.9	17.9	12.9	22.1	12.5
- Unknown	4.3	3.0	4.3	2.6	4.4
Elixhauser Comorbidities, %					
- Congestive heart failure	3.8	12.7	3.7	10.5	3.5
- Cardiac arrhythmias	5.6	15.7	5.5	14.9	5.1
- Valvular disease	1.0	1.6	1.0	1.8	0.9
- Pulmonary circulation disorders	1.0	4.3	1.0	3.2	0.9
- Peripheral vascular disorders	3.0	7.1	3.0	8.7	2.7
- Hypertension (uncomplicated)	12.2	23.9	12.1	29.7	11.3
- Hypertension (with complications)	0.3	1.6	0.3	1.1	0.3
- Paralysis	1.1	7.9	1.1	5.3	0.9
- Neurodegenerative disorders	3.2	8.7	3.2	8.6	3.0
- COPD	5.4	12.2	5.3	11.7	5.1
- Diabetes (uncomplicated)	8.6	15.5	8.5	16.8	8.2
- Diabetes (with complications)	7.7	23.6	7.5	21.8	6.9
- Hypothyroidism	1.3	2.2	1.3	2.3	1.2
- Renal failure	2.9	6.8	2.9	8.1	2.7
- Liver disease	1.4	4.9	1.3	3.9	1.2
- Peptic ulcer disease, no bleeding	0.6	3.0	0.5	1.6	0.5
- AIDS/HIV	0.2	0.3	0.2	0.5	0.2
- Lymphoma	1.2	4.3	1.2	4.4	1.1
- Metastatic cancer	4.4	4.9	4.4	7.2	4.3
- Solid tumor without metastasis	11.2	14.1	11.2	18.9	10.8
- Rheumatoid arthritis	0.9	1.9	0.9	1.6	0.8
- Coagulopathy	1.2	5.2	1.2	3.9	1.1
- Obesity	1.6	2.7	1.5	2.1	1.5
- Fluid and electrolyte disorders	1.2	4.1	1.2	4.3	1.1
- Blood loss anemia	0.2	0.5	0.2	0.4	0.2
- Deficiency anemia	1.2	2.7	1.2	3.0	1.1
- Alcohol abuse	2.0	6.2	1.9	4.1	1.8
- Drug abuse	1.1	2.7	1.1	2.0	1.1
- Psychosis	0.9	1.4	0.9	1.7	0.9
- Depression	2.2	5.4	2.1	3.8	2.1
Elixhauser Comorbidity Score, median (IQR)	0 (0–4)	4 (0–11)	0 (0–4)	5 (0–11)	0 (0–4)

Data Source: Ottawa Hospital Data Warehouse

*Abbreviations*: *AIDS/HIV* Acquired Immune Deficiency Syndrome/Human Immunodeficiency Virus, *COPD* Chronic Obstructive Pulmonary Disease, *IQR* Inter-quartile Range

High cost groups were more likely to have multiple hospital encounters compared to non-high cost patients (Table 2). However, over half of the high cost patients accumulated costs from a single event (top 1; 52.3 % and top 5; 54.3 %). Median total days spent in hospital were markedly higher for high cost patients compared to non-high cost patients (52 days versus 3 days). The proportion of high cost patients with at least one ICU admission was also larger than the non-high cost comparator (48.4 versus 7.1 %) and high cost patients were more likely to have ALC days during their hospital encounter (33.2 versus 2.7 %). These values increased for those that were in the top 1 % high cost group with almost three-quarters (73.4 %) having at least one ICU day and 37.1 % spending one or more hospital days in an ALC setting. Finally, in-hospital mortality and 30-day all-cause readmission were higher in the high cost cohorts. Approximately 20 % of high cost patients died in hospital and over 30 % were readmitted. These proportions were higher for the extremely high cost cohort.

**Table 2:** Number of hospitalizations and length of stay among high cost inpatients

		Top 1 %		Top 5 %	
Variable	All	High Cost	Non High Cost	High Cost	Non High Cost
	(*n* = 36,892)	(*n* = 369)	(*n* = 36,523)	(*n* = 1,844)	(*n* = 35,048)
Number of hospitalizations, %					
1	86.5	52.3	86.9	54.3	88.2
2	9.9	19.5	9.8	22.6	9.2
3	2.3	12.2	2.2	11.8	1.8
≥ 4	1.3	16.0	1.1	11.3	0.8
Total days in hospital, median (IQR)	3 (2–7)	91 (62–129)	3 (2–7)	52 (35–77)	3 (2–6)
Acute days in hospital, median (IQR)	3 (2–7)	68 (48–99)	3 (2–7)	40 (27–58)	3 (2–6)
≥1 ALC day, %	4.2	37.1	3.9	33.2	2.7
ALC days in hospital, median (IQR)^a^	13 (5–34)	43 (17–126)	12 (5–30)	31 (11–68)	8 (4–20)
≥1 ICU admission, %	9.2	73.4	8.6	48.4	7.1
ICU days in hospital, median (IQR)^b^	4 (1–10)	26 (14–39)	3 (1–9)	14 (8–25)	2 (1–6)
In-hospital mortality, %	4.6	21.7	4.4	20.4	3.8
30-day all-cause readmission, %^c^	(*n* = 35,067) 6.8	(*n* = 310) 35.8	(*n* = 34,757) 6.5	(*n* = 1548) 31.9	(*n* = 33,519) 5.6

Data Source: Ottawa Hospital Data Warehouse

*Note*: Total length of stay is Acute days + ALC days. Acute days include ICU days

*Abbreviations*: *ALC* Alternate Level of Care, *ICU* Intensive Care Unit, *IQR* Inter-quartile Range

^a^Among patients with at least 1 ALC day

^b^Among patients with an ICU admission

^c^Among patients eligible for hospital readmission (excludes patients that died in hospital prior to discharge)

Given the large proportion of high cost patients that accumulated cost from a single encounter, admission type and most responsible diagnosis for these events were explored (Additional file 3). Approximately 60 % of extremely high cost patients with a single hospitalization (*n* = 193) were emergency admissions. The most frequent diagnoses for these patients were acute respiratory failure, sepsis, or low birth-weight delivery with average length of stays often exceeding 60 days. Similar findings were observed in high cost patients in the top 5 % percentile with a single encounter (*n* = 1002).

### Acute care spending

Approximately $320 million in direct inpatient spending was accumulated over the study period (Table 3). The top 1 and 5 % of patients accounted for 15.3 and 38.1 % of total spending. The average (SD) cost per patient was $8,716 (17,646). This was markedly higher for those in the top 1 and 5 % ($133,190 (57,739) and $66,407 (44,326) respectively). Nursing costs accounted for almost half of the total spending within the entire study cohort (45.7 %). While nursing costs remained a large component of total spending within high cost groups, over one third (34 %) of expenditures among extremely high cost patients were related to special care unit resources (ICU care). High cost groups also had higher proportional spending on pharmacy and health professional services.

**Table 3:** Concentration of direct inpatient spending and resource utilization among high cost patients

	All	Top 1 %	Top 5 %
	(*n* = 36,892)	(*n* = 369)	(*n* = 1844)
Direct Inpatient Spending ($)	321,556,160	49,147,104	122,454,872
Percentage of total inpatient spending (%)	100.0	15.3	38.1
Average direct cost per patient $ (SD)	8,716 (17,646)	133,190 (57,739)	66,407 (44,326)
Resource Utilization (proportion of direct inpatient spending) %			
Endoscopy	0.3	0.3	0.3
Food Services	2.5	2.3	2.6
Health Professionals	5.0	9.4	7.7
Imaging	3.2	2.8	3.2
Laboratory	7.2	4.9	5.5
Nursing	45.7	33.7	40.2
Operating Room	8.7	2.0	3.1
Operating Room Implants	4.2	1.5	2.0
Pharmacy	6.7	8.7	8.4
Post-anesthesia Care Unit	2.1	0.4	0.7
Special Care Unit	14.4	34.0	26.3
Direct inpatient spending attributable to ALC days $(%)	$15,789,200 (4.9 %)	$3,985,800 (8.1 %)	$11,140,500 (9.1 %)

Data Source: Case Costing System within Ottawa Hospital Data Warehouse

*Abbreviations*: *ALC* Alternate Level of Care, *SD* Standard Deviation

### Preventable acute care spending

Among all inpatients aged <75 years, the proportion of patients with a hospital encounter deemed ambulatory care sensitive was 2.9 % (95 % CI: 2.7–3.2) (Table 4). This was twice as high for high cost patients relative to non-high cost patients (6.0 %; 95 % CI: 4.5–7.5 versus 2.8 %; 95 % CI: 2.6–3.0) (*χ*^2^*p* < 0.001). While the absolute values are small, the most common condition-specific ACS hospitalizations related to COPD, CHF/Pulmonary edema, and diabetes. These proportions were consistently higher among the high cost cohort relative to their non-high cost comparator.

**Table 4:** Proportion of patients with an ambulatory care sensitive hospital encounter

	All patients		High Cost (Top 5 %)		Non-High Cost (Lower 95 %)	
	(*n* = 23,620)		(*n* = 968)		(*n* = 22,652)	
	*N*	% (95 % CI)	*N*	% (95 % CI)	*N*	% (95 % CI)
Any ACSC Hospitalization	695	2.9 (2.7–3.2)	58	6.0 (4.5–7.5)	637	2.8 (2.6–3.0)
Condition-specific Hospitalization						
- Epilepsy	78	0.3 (0.2–0.4)	7	0.7 (0.2–1.3)	71	0.3 (0.2–0.4)
- COPD	266	1.1 (0.9–1.3)	23	2.4 (1.4–3.3)	243	1.1 (0.9–1.2)
- Asthma	47	0.2 (0.1–0.3)	2	0.2 (0.0–0.5)	45	0.2 (0.1–0.3)
- CHF/Pulmonary Edema	135	0.6 (0.5–0.7)	12	1.3 (0.5–2.0)	123	0.6 (0.4–0.7)
- Hypertension	13	0.1 (0.0–0.1)	0	0 (0–0)	13	0.1 (0.0–0.1)
- Angina	37	0.2 (0.1–0.3)	1	0.1 (0.0–0.3)	36	0.2 (0.1–0.3)
- Diabetes	112	0.5 (0.4–0.6)	13	1.3 (0.6–2.1)	99	0.4 (0.3–0.5)

Data Source: Ottawa Hospital Data Warehouse

*Abbreviations*: *ACSC* Ambulatory Care Sensitive Condition, *CHF* Congestive Heart Failure, *CI* Confidence Interval

**Fig. 1:**
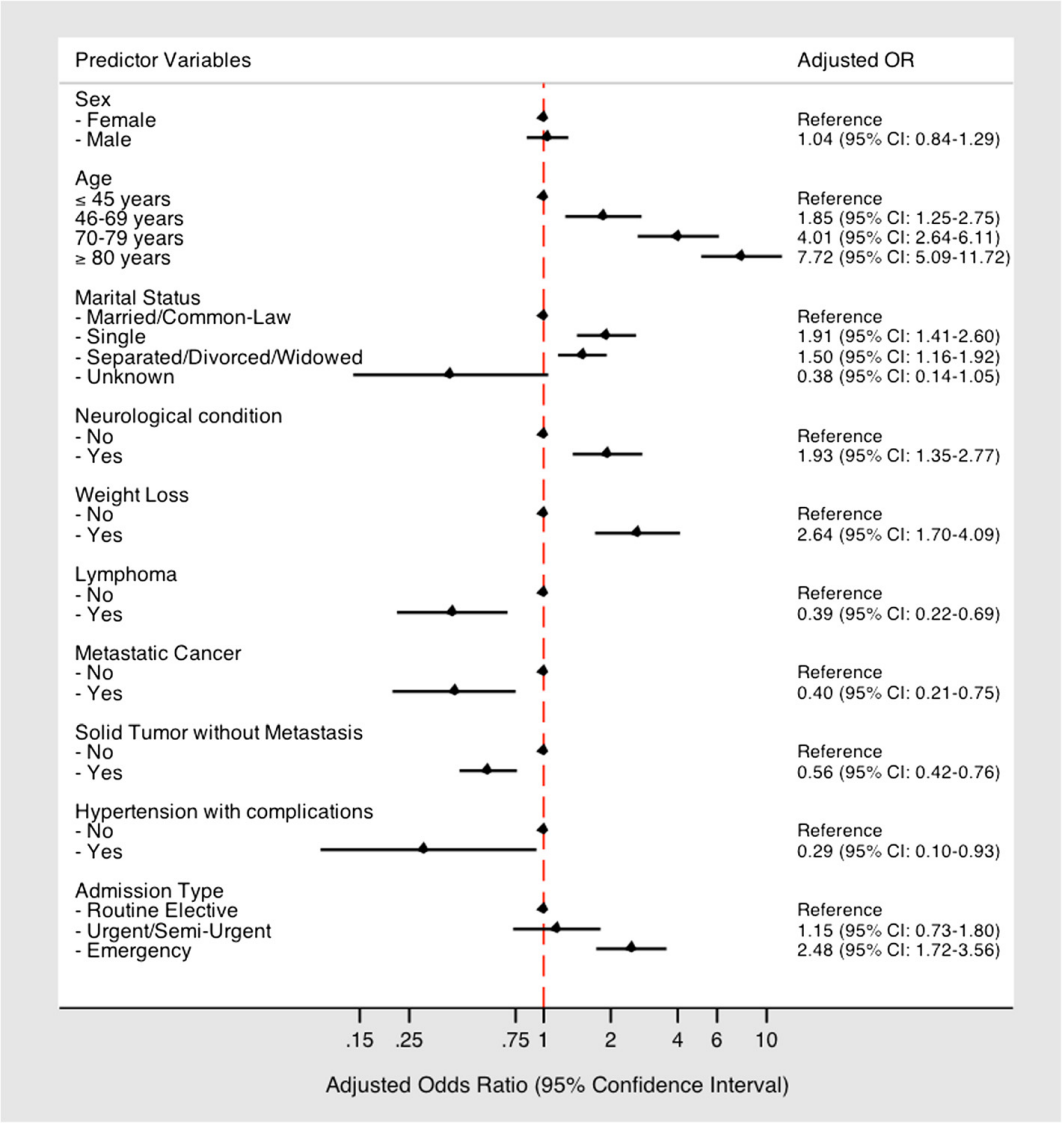
Predictors of one or more ALC days among high cost inpatients

Approximately 9 % of total direct inpatient spending among high cost patients was attributable to ALC days (Table 3). This value was significantly higher than the proportion of spending attributed to ALC days within non-high cost comparators (4.9 %) (*χ*^2^*p* < 0.001). Based on our regression analysis, the odds of having one or more ALC days among high cost inpatients increased with age (Fig. 1). Patients 80 years of age or older were seven times more likely to have ALC days compared to patients ≤45 years of age (OR: 7.72; 95 % CI: 5.09–11.72). The odds of having ALC days were higher for single patients, widowed/divorced or separated patients (compared to married patients), and those with neurological comorbidity, while high cost patients with cancer-related comorbidities were less likely. Furthermore, patients with emergency admissions were two times more likely to have ALC days compared to those with elective admissions (OR: 2.47; 95 % CI: 1.72–3.56).

## Discussion

In this cohort of patients from a Canadian academic acute care centre, we found that a small proportion of patients accounted for the majority of hospital spending. While the clinical characteristics of these patients varied substantially, high cost patients were often elderly, medically complex, and accumulated high costs from a single hospital encounter. These costs were generated predominantly from nursing care and from special units such as intensive care. This suggests a limit in the ability to reduce this spending, as it appears that these patients are simply very sick and therefore require expensive care. On the other hand, the proportion of acute care spending deemed potentially preventable amongst this high-risk group was not inconsequential. Almost 50 % of these patients had multiple admissions (many representing readmissions), 6 % of them had an encounter that was deemed ambulatory care sensitive, and almost 10 % of total direct inpatient spending was attributable to difficulties transitioning high cost patients to alternate care settings. While these appear to be areas to focus, there would of course be offsetting costs for providing care elsewhere.

Our findings suggest some opportunities for improved value for money but should also be viewed cautiously. It is clear that in a hospital setting there is significant concentration of spending within a small group of patients, who on the whole were older and sicker than all other patients. What is not entirely clear is whether we can use this information to guide improvements in services or whether other avenues of potentially preventable inpatient spending should be considered (e.g. patient safety indicators such as adverse events in hospital). We did find several patterns suggesting a direction for improvement. The most important finding, in our view, is that over a third of high cost patients had at least one ALC day and that spending on this level of care attributed to almost 10 % of the overall spending in this group. It is likely that difficulty finding discharge locations is the cause of the spending, as these values are higher than those observed in our overall population, which are comparable to the general Canadian population, ranging from 2–7 % [24]. It is also likely that there are diverse sets of inter-related social and medical factors contributing to this situation that would require additional exploration through a mixed-methods approach. Regardless of cause, this is not acceptable in an academic hospital that is supposed to be reserved for providing the most complex advanced tertiary care. Importantly, we were able to identify specific patient characteristics that increased the likelihood of incurring ALC day among the most costly inpatients including increased age, marital status and specific comorbid conditions. Improving transitions of care for those at greatest risk of having ALC days may therefore represent an avenue where costs could be contained. However, this relies on the need for improved models of community-based care for complex medical patients and an exploration of the potential barriers that impede efficient transitions out of acute care [27]. Whether the costs associated with these alternative approaches are less than what is spent in hospital remains to be determined.

Similar to other investigators, we also found that there were significant admissions for so-called ambulatory care sensitive conditions. Joynt et al. recently quantified preventable acute care spending among high cost Medicare beneficiaries and found that 15.8 % of hospitalizations were attributable to preventable causes [5]. This rate is almost three times higher than we observed. This opens up the possibility that our publicly funded system, providing universal access to primary care, may have significant downstream benefits. In contrast, while Joynt and colleagues did not explicitly report on costs attributable to difficulty discharging patients, ALC-related spending may simply be a Canadian health system phenomenon. There is therefore a need for comparative research across health care models and a determination of system factors that may create inefficiencies in acute care spending at either end of the hospital encounter. Similar to our results, they found that COPD and CHF were two of the most common diagnoses for preventable hospitalizations. Improving outpatient management for patients with chronic disease remains a priority within health care. Various outpatient interventions have been shown to reduce the risk of re-hospitalization, and in turn inpatient costs, in the setting of specific chronic conditions; most notably COPD [28–30]. Whether these interventions can be put in place to reduce ACS hospitalizations for medically complex patients, with multiple comorbidities, remains to be determined and represents an area of future research. Despite this, it is important to interpret these findings in absolute terms. The fact that only 6 % of hospital encounters were deemed ambulatory care sensitive suggests it may be difficult to target specific sub-groups of high cost, medically complex patients to intervene upon.

Our study has a number of strengths, including a large sample of all patients referred to a typical acute care hospital within Canada. The use of detailed clinical and costing information also provides us with valuable insight into the characteristics of high cost patients and the specific hospital resources they use. However, our study should also be interpreted in light of its limitations. First, we did not assess spending outside of the hospital setting. While inpatient spending makes up a large proportion of health care spending, there is likely a number of different “high cost” profiles based on spending outside of the acute care setting. Specifically, our estimated costs do not include physician expenditures, which would result in an underestimate of total spending. However, the relative contribution of physician costs to the total expenditures is fairly small among high system users [31]. Second, our inclusion criterion was limited to admissions that occurred within one fiscal year resulting in potential misclassification of high cost patients based on differential follow-up times. In this specific scenario, it is likely to underestimate the total number of high cost patients identified but have minimal impact on the overall study findings including the varied clinical profiles of high cost patients and that these costs were often attributed to a single hospital encounter. Third, our estimates of preventable hospitalizations using the ACS algorithm should be interpreted with caution as they exclude a proportion of patients identified as high cost based on our operational definition (i.e. newborns and patients >75 years). This may underestimate the total proportion of events deemed potentially avoidable. Our estimates also represent a spectrum of preventability that requires exploration into other aspects of patient care, including supply and use of outpatient services. It also requires the inclusion of system/provider-level metrics of primary care performance in the analysis of ACS hospitalization as well as additional patient-level factors related to chronic disease progression or severity. Finally, this study was limited to a single Canadian tertiary care setting and may not be generalizable to other jurisdictions with variations in primary care delivery, capacity to care for patients within the community, and patient characteristics. While the majority of residents of Ottawa and surrounding areas are served by the 2 acute care facilities included within our analysis, there are other inpatient facilities that are not captured within the Ottawa Hospital Data Warehouse. Although it is possible that a proportion of high and non-high users may be misclassified based on inpatient use within and outside of those reported to the Data Warehouse, we believe this is likely to be non-differential in nature.

## Conclusions

In summary, within a population of high cost inpatients, we found that an important proportion of costs appear to be related to preventable hospital days – resulting from ambulatory care sensitive and repeat encounters *and* more importantly difficulty discharging patients. While improving care and mitigating costs remain paramount, these results suggest there are opportunities to lower costs for these patients through better outpatient management and coordination of care. Future work is needed to explore the potential barriers impeding efficient transitions out of acute care for complex medical patients. There is also a need to develop population-based estimates on preventable acute care spending and to explore how variations in coordination of care and care delivery models influence these estimates.

### Ethical approval and consent to participate

This study was approved by the Ottawa Health Sciences Network Research Ethics Board and granted waiver of patient consent.

### Consent for publication

Not applicable.

### Availability of data and material

The datasets supporting the conclusions of this article are housed within the Ottawa Hospital Data Warehouse and are not publically available.

## Additional files

Additional file 1: Resource-specific direct cost categories. (DOCX 48 kb) Additional file 2: Ambulatory Care Sensitive Conditions (ACSC) algorithm. (DOCX 79 kb) Additional file 3: Most responsible diagnoses observed in high cost patients with a single hospital encounter. (DOCX 69 kb)

## Abbreviations

ACS: ambulatory care sensitive
ALC: alternate level of care
CHF: congestive heart failure
CI: confidence interval
CIHI: Canadian Institute for Health Information
COPD: chronic obstructive pulmonary disease
ICD-10-CA: International Statistical Classification of Diseases and Related Health Problems, 10^th^ revision Canada
ICU: intensive care unit
IQR: inter-quartile range
OR: odds ratio
SD: standard deviation
