# Supplementary material for: Clinical characteristics and preventable acute care spending among a high cost inpatient population
Source: BMC Health Serv Res. 2016 May 4;16:165. doi: 10.1186/s12913-016-1418-2 (PMC4855849; doi:10.1186/s12913-016-1418-2)
Supplement: Additional file 1: — Resource-specific direct cost categories. (DOCX 48 kb) [file 12913_2016_1418_MOESM1_ESM.docx]

Additional File 1. Resource-specific direct cost categories

| - Endoscopy  - Food services  - Health Professionals (including occupational therapy, speech and language pathology, social work,  recreational therapy, physiotherapy, respiratory services)  - Imaging (including electro-diagnostics and non-invasive vascular imaging)  - Laboratory  - Nursing costs (including ward, non-ward (emergency, hemodialysis), and intensive care unit)  - Operating Room  - Operating Room Implants  - Pharmacy  - Post-anesthetic Care Unit  - Special Care Unit (includes neonatal ICU, coronary ICU, medical/surgical ICU) |
| --- |

Data Source: Ottawa Hospital Data Warehouse – Case Costing System

- Categories adapted from the Ontario Quality Based Procedures Groupings
