# Supplementary material for: Clinical characteristics and preventable acute care spending among a high cost inpatient population
Source: BMC Health Serv Res. 2016 May 4;16:165. doi: 10.1186/s12913-016-1418-2 (PMC4855849; doi:10.1186/s12913-016-1418-2)
Supplement: Additional file 2: — Ambulatory Care Sensitive Conditions (ACSC) algorithm. (DOCX 79 kb) [file 12913_2016_1418_MOESM2_ESM.docx]

Additional File 2. Ambulatory Care Sensitive Conditions (ACSC) algorithm

| Condition | Most Responsible Diagnosis (ICD-10-CA) |
| --- | --- |
| Epilepsy | G40, G41 |
| COPD | J41, J42, J43, J44, J47 |
| Asthma | J45 |
| Heart failure/Pulmonary Edema* | I50, J81 |
| Hypertension* | I10.0, I10.1, I11 |
| Angina* | I20, I23.82, I24.0, I24.8, I24.9 |
| Diabetes | E10.0^^, E10.1^^, E10.63, E10.64, E10.9^^, E11.0^^, E11.1^^, E11.63, E11.64, E11.9^^, E13.0^^, E13.1^^, E13.63, E13.64, E13.9^^, E14.0^^, E14.1^^, E14.63, E14.64, E14.9^^ |

Abbreviations: CCI=Canadian Classification of Health Interventions; COPD=Chronic Obstructive Pulmonary Disease; ICD-10-CA=International Statistical Classification of Diseases and Related Health Problems – 10^th^ Revision – Canada.

Patient Exclusion criteria:

1. Death before discharge
2. Individuals age 75 years and older
3. Admission category recorded as newborn or stillbirth

* List of cardiac procedure codes for exclusion:

CCI: 1HA58, 1HA80, 1HA87, 1HB53, 1HB54, 1HB55, 1HB87, 1HD53, 1HD54, 1HD55, 1HH59, 1HH71, 1HJ76, 1HJ82, 1HM57, 1HM78, 1HM80, 1HN71, 1HN80, 1HN87, 1HP76, 1HP78, 1HP80, 1HP82, 1HP83, 1HP87, 1HR71, 1HR80, 1HR84, 1HR87, 1HS80, 1HS90, 1HT80, 1HT89, 1HT90, 1HU80, 1HU90, 1HV80, 1HV90, 1HW78, 1HW79, 1HX71, 1HX78, 1HX79, 1HX80, 1HX83, 1HX86, 1HX87, 1HY85, 1HZ53 rubric (except 1HZ53LAKP), 1HZ55 rubric (except 1HZ55LAKP), 1HZ56, 1HZ57, 1HZ59, 1HZ80, 1HZ85, 1HZ87, 1IF83, 1IJ50, 1IJ55, 1IJ57, 1IJ76, 1IJ86, 1IJ80, 1IK57, 1IK80, 1IK87, 1IN84, 1LA84, 1LC84, 1LD84, 1YY54LANJ

Data Source: Canadian Institute for Health Information (CIHI)
