# Supplementary material for: Clinical characteristics and preventable acute care spending among a high cost inpatient population
Source: BMC Health Serv Res. 2016 May 4;16:165. doi: 10.1186/s12913-016-1418-2 (PMC4855849; doi:10.1186/s12913-016-1418-2)
Supplement: Additional file 3: — Most responsible diagnoses observed in high cost patients with a single hospital encounter. (DOCX 69 kb) [file 12913_2016_1418_MOESM3_ESM.docx]

Additional File 3. Most responsible diagnoses observed in high cost patients with a single hospital encounter

| High Cost inpatients with one encounter | Admission Type | Most frequent MRDX codes | Average LOS  Mean (SD), Range |
| --- | --- | --- | --- |
| Top 1 % (n=193) | Elective (5.7%)  Emergency (59.1%)  Newborn (9.3%)  Urgent (19.7%)  Same day admit (6.2%) | - A41.9 (Sepsis – unspecified) (7.8%) - J96.0 (Acute respiratory failure) (6.7%) - P07.0 (Extremely low birth weight) (5.7%) | 62 (37), 23-161  77 (53), 9-226  75 (32), 4-122 |
| Top 5% (n=1002) | Elective (6.9%)  Emergency (65.1%)  Newborn (8.1%)  Urgent (15.8%)  Same day admit (4.2%) | - P07.1 (Low birth weight) (4.4%) - A41.9 (Sepsis – unspecified) (3.5%) - J96.0 (Acute respiratory failure) (2.8%) - P07.0 (Extremely low birth weight) (2.8%) - R64 (Cachexia (wasting syndrome) (1.9%) - J18.9 (Pneumonia - unspecified) (1.8%) - I71.4 (Abdominal aortic aneurysm without rupture) (1.7%) - S066 (Traumatic subarachnoid hemorrhage) (1.4%) - J44.0 (COPD) (1.2%) | 46 (15), 14-93  42 (34), 4-161  51 (47), 9-226  62 (28), 4-122  107 (47), 14-202  36 (52), 1-278  17 (14), 2-49  74 (28), 33-118  33 (26), 2-105 |

Abbreviations: COPD=Chronic Obstructive Pulmonary Disease; LOS=Length of Stay; MRDX=Most Responsible Diagnosis; SD=Standard Deviation

Data Source: Ottawa Hospital Data Warehouse
